# A contrast-enhanced CT-based radiomic nomogram for the differential diagnosis of intravenous leiomyomatosis and uterine leiomyoma

**DOI:** 10.3389/fonc.2023.1239124

**Published:** 2023-08-23

**Authors:** Jiang Shao, Chaonan Wang, Keqiang Shu, Yan Zhou, Ninghai Cheng, Zhichao Lai, Kang Li, Leyin Xu, Junye Chen, Fenghe Du, Xiaoxi Yu, Zhan Zhu, Jiaxian Wang, Yuyao Feng, Yixuan Yang, Xiaolong Liu, Jinghui Yuan, Bao Liu

**Affiliations:** ^1^ Department of Vascular Surgery, Peking Union Medical College Hospital, Peking Union Medical College, Chinese Academy of Medical Science, Beijing, China; ^2^ Plastic Surgery Hospital, Chinese Academy of Medical Science, Peking Union Medical College, Beijing, China; ^3^ Eight-year Program of Clinical Medicine, Peking Union Medical College Hospital, Peking Union Medical College, Chinese Academy of Medical Sciences, Beijing, China; ^4^ National Clinical Research Center for Obstetric & Gynecologic Diseases, Department of Obstetrics and Gynecology, Peking Union Medical College Hospital, Peking Union Medical College, Chinese Academy of Medical Sciences, Beijing, China; ^5^ State Key Laboratory of Medical Molecular Biology, Institute of Basic Medical Sciences, Chinese Academy of Medical Sciences, Department of Pathophysiology, Peking Union Medical College, Beijing, China; ^6^ Peking Union Medical College, MD Program, Beijing, China

**Keywords:** intravenous leiomyomatosis, contrast-enhanced CT, radiomics, preoperative differential, nomogram

## Abstract

**Objective:**

Uterine intravenous leiomyomatosis (IVL) is a rare and unique leiomyoma that is difficult to surgery due to its ability to extend into intra- and extra-uterine vasculature. And it is difficult to differentiate from uterine leiomyoma (LM) by conventional CT scanning, which results in a large number of missed diagnoses. This study aimed to evaluate the utility of a contrast-enhanced CT-based radiomic nomogram for preoperative differentiation of IVL and LM.

**Methods:**

124 patients (37 IVL and 87 LM) were retrospectively enrolled in the study. Radiomic features were extracted from contrast-enhanced CT before surgery. Clinical, radiomic, and combined models were developed using LightGBM (Light Gradient Boosting Machine) algorithm to differentiate IVL and LM. The clinical and radiomic signatures were integrated into a nomogram. The diagnostic performance of the models was evaluated using the area under the curve (AUC) and decision curve analysis (DCA).

**Results:**

Clinical factors, such as symptoms, menopausal status, age, and selected imaging features, were found to have significant correlations with the differential diagnosis of IVL and LM. A total of 108 radiomic features were extracted from contrast-enhanced CT images and selected for analysis. 29 radiomics features were selected to establish the Rad-score. A clinical model was developed to discriminate IVL and LM (AUC=0.826). Radiomic models were used to effectively differentiate IVL and LM (AUC=0.980). This radiological nomogram combined the Rad-score with independent clinical factors showed better differentiation efficiency than the clinical model (AUC=0.985, p=0.046).

**Conclusion:**

This study provides evidence for the utility of a radiomic nomogram integrating clinical and radiomic signatures for differentiating IVL and LM with improved diagnostic accuracy. The nomogram may be useful in clinical decision-making and provide recommendations for clinical treatment.

## 1 Introduction

Intravenous leiomyomatosis (IVL) is a rare benign type of uterine leiomyoma. Although histologically benign, it can spread to the extrauterine venous system or even the heart and pulmonary arterial system (1, 2). The current information on IVL mainly comes from case reports and case series, and its clinical presentation is nonspecific and may lead to right heart obstruction, pulmonary embolism and even sudden death (3). The development of IVL is insidious, and the clinical symptoms and pathological imaging features lack specificity and can cause serious consequences, especially in patients presenting with cardiac symptoms.

In addition, the pathological presentation of IVL is the same as that of common uterine leiomyoma (LM), and it may be difficult for pathologists to distinguish it from LM in patients with primary LM combined with IVL, especially if the lesions are confined to the uterus without invasion of the extrauterine veins. Some patients were only diagnosed with IVL after a previous hysterectomy to remove a primary uterine tumor. Pathological tissue findings of invasion of the parauterine veins may be a marker for IVL diagnosis. As a result, IVL is often underestimated due to the ease of misdiagnosis and the lack of specific identifying biomarkers.

The imaging presentation of IVL depends on the location and extent of its involvement. Typical imaging methods for the diagnosis of IVL include ultrasonography, computed tomography (CT) and magnetic resonance imaging (MRI). When a mass is confined to the pelvis, it is difficult to completely distinguish between IVL and LM on the basis of traditional radiology alone unless it has invaded the extrauterine vessels and is growing invasively (4). Radiomics refers to quantitative methods of extracting image features from conventional radiographic images and analyzing the data to create models with features to aid in diagnosis, prediction and prognosis (5). Previous studies have demonstrated the value of radiomic features as imaging predictors that can be used to treat and diagnose various types of tumors (6). A study applied a radiomic model generated from features extracted from the region of interest covering the uterus with good diagnostic performance for uterine sarcomas and leiomyomas (7). However, no research has been performed to determine whether contrast-enhanced CT-based radiomics can be used to differentiate IVL and LM.

Therefore, this study aimed to use radiomics features extracted from clinically acquired abdominal pelvic CT scans to predict whether LM patients have IVL features prior to treatment.

## 2 Materials and methods

### 2.1 Patients

**Figure 1:**
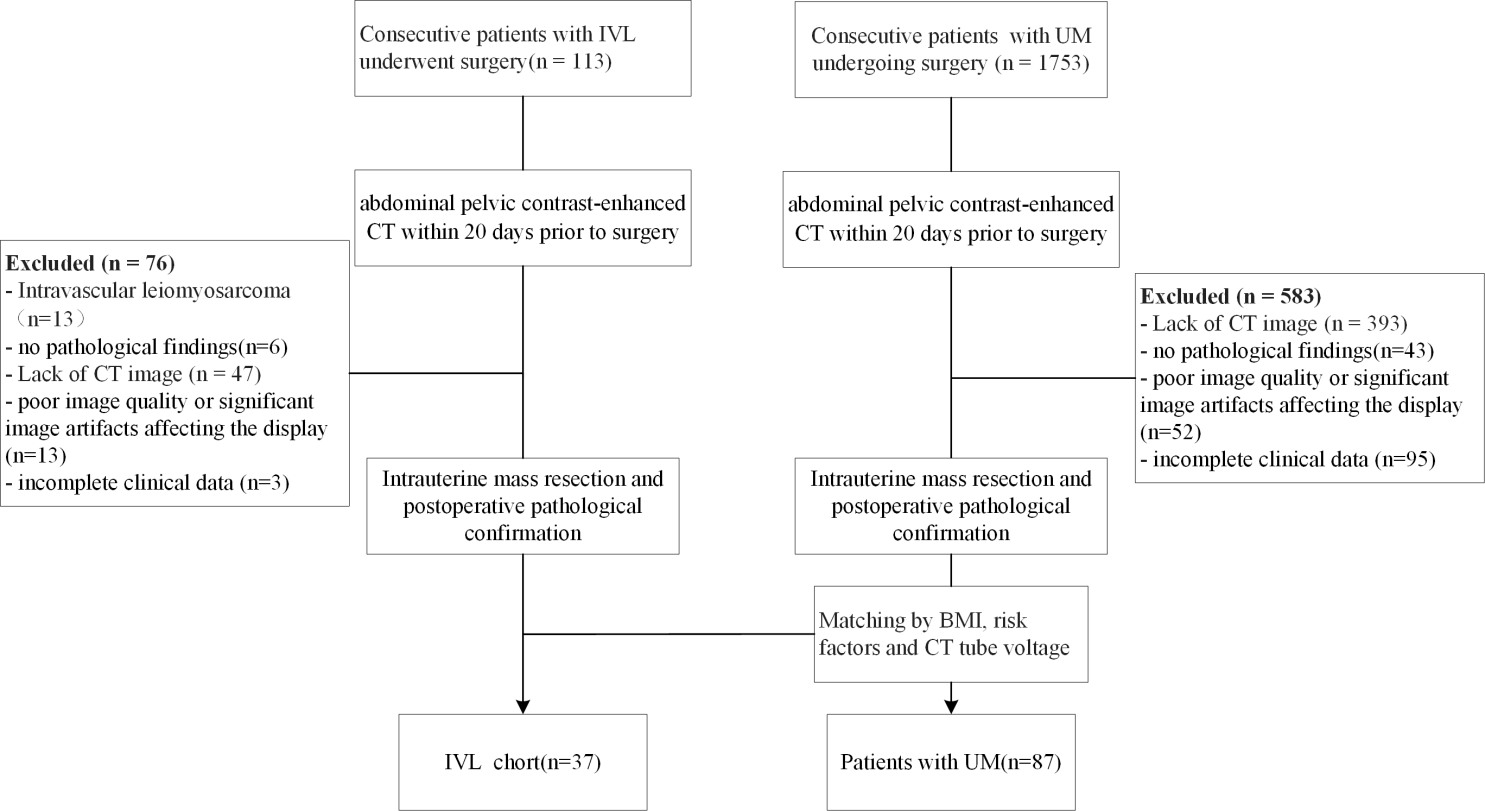
Flow chart demonstrating the inclusion and exclusion criteria for the study participants with IVL and UM. IVL, intravenous leiomyomatosis; UM, uterine leiomyoma; BMI, body mass index; CTA, computed tomography.

The Peking Union Medical College Hospital (PUMCH) ethics committee approved the study and waived informed consent from the patients (No. JS-2964). We reviewed the PUMCH surgical database. Patients who underwent gynecologic surgery between January 2011 and December 2020 were pathologically confirmed to have IVL. The inclusion criteria were as follows: 1) surgically and pathologically confirmed IVL or LM; 2) abdominal pelvic contrast-enhanced CT within the 20 days prior to gynecologic surgery; and 3) no relevant treatment prior to CT examination. The exclusion criteria were as follows: 1) no pathological findings, 2) poor image quality or significant image artifacts affecting the visualization, 3) incomplete clinical data, 4) intravascular leiomyosarcoma. 5) and a lack of CT images. Patients with uterine LM were matched to those who underwent surgery for uterine neoplasms by BMI, risk factors, and CT tube voltage. Ultimately, CT results from 124 patients (37 IVL and 87 LM) were included in the study. **Figure 1** shows the flow chart of patient enrollment.

### 2.2 CT scan protocol

Patients who underwent contrast-enhanced CT examinations of the abdomen and pelvis were examined using GE Discovery CT (GE Medical, Piscataway, NJ, USA) or Somatom Definition Flash CT (Siemens Medical Solutions, Germany). All patients underwent thin-slice image scanning using a soft tissue algorithm, and CT images were obtained for the arterial (30 seconds postinjection), venous (60 seconds postinjection), and delayed (120 seconds postinjection) phases. The scanning parameters were as follows: tube voltage, 120 kV with automatic tube current modulation initiated; collimation, Somatom Definition Flash CT 128 × 0.6 mm, GE Discovery CT 64 × 0.6 mm; slice thickness, 0.625-1 mm; slice interval, 0.625-1 mm.

### 2.3 Image segmentation

The target of image segmentation is the intrauterine mass. When there were multiple masses in the uterus, the largest mass was chosen as the region of interest (ROI). Image segmentation was performed independently by two radiologists with extensive experience in gynecologic tumor imaging diagnosis. They were blinded to the patients’ histopathology. One of the radiologists (radiologist A, with 7 years of experience in diagnostic imaging of gynecologic tumors) manually drew the ROI slice by slice using the open-source software 3D Slicer 4.11.0 (https://www.slicer.org/) (8). Another radiologist (radiologist B, with 10 years of experience in diagnostic imaging of gynecologic tumors) reviewed all ROIs manually segmented by radiologist A.

### 2.4 Data preprocessing

The dataset was randomly assigned in a 3:1 ratio to either the training dataset or test dataset. All cases in the training dataset were used to train the predictive model, while cases in the test dataset were used to independently evaluate the model’s performance. Medical volumes are common with heterogeneous voxel spacing because of different scanners or different acquisition protocols. Such spacing refers to the physical distance between two pixels in an image. Spatial normalization is often employed to reduce the effect of voxel spacing variation. The fixed resolution resampling method was used in our experiment to handle the aforementioned problems. All images were resampled to a voxel size of 3*3*3 mm to standardize the voxel spacing. Finally, the data were standardized using z score standardization (zero-mean normalization).

### 2.5 Radiomics feature extraction

The handcrafted features can be divided into three groups: (I) geometry, (II) intensity and (III) texture. The geometric features describe the three-dimensional shape characteristics of the tumor. The intensity features describe the first-order statistical distribution of the voxel intensities within the tumor. The texture features describe the patterns or the second- and high-order spatial distributions of the intensities. Here, the texture features were extracted using several different methods, including the gray-level cooccurrence matrix (GLCM), gray-level run length matrix (GLRLM), gray level size zone matrix (GLSZM) and neighborhood gray-tone difference matrix (NGTDM) methods. A total of 107 categories of handcrafted features were extracted, including 18 geometry features, 14 intensity features, and 75 texture features. All handcrafted features were extracted with an in-house feature analysis program implemented using Pyradiomics (http://pyradiomics.readthedocs.io).

### 2.6 Radiomics feature selection

#### 2.6.1 Intraclass correlation coefficient

**Figure 2:**
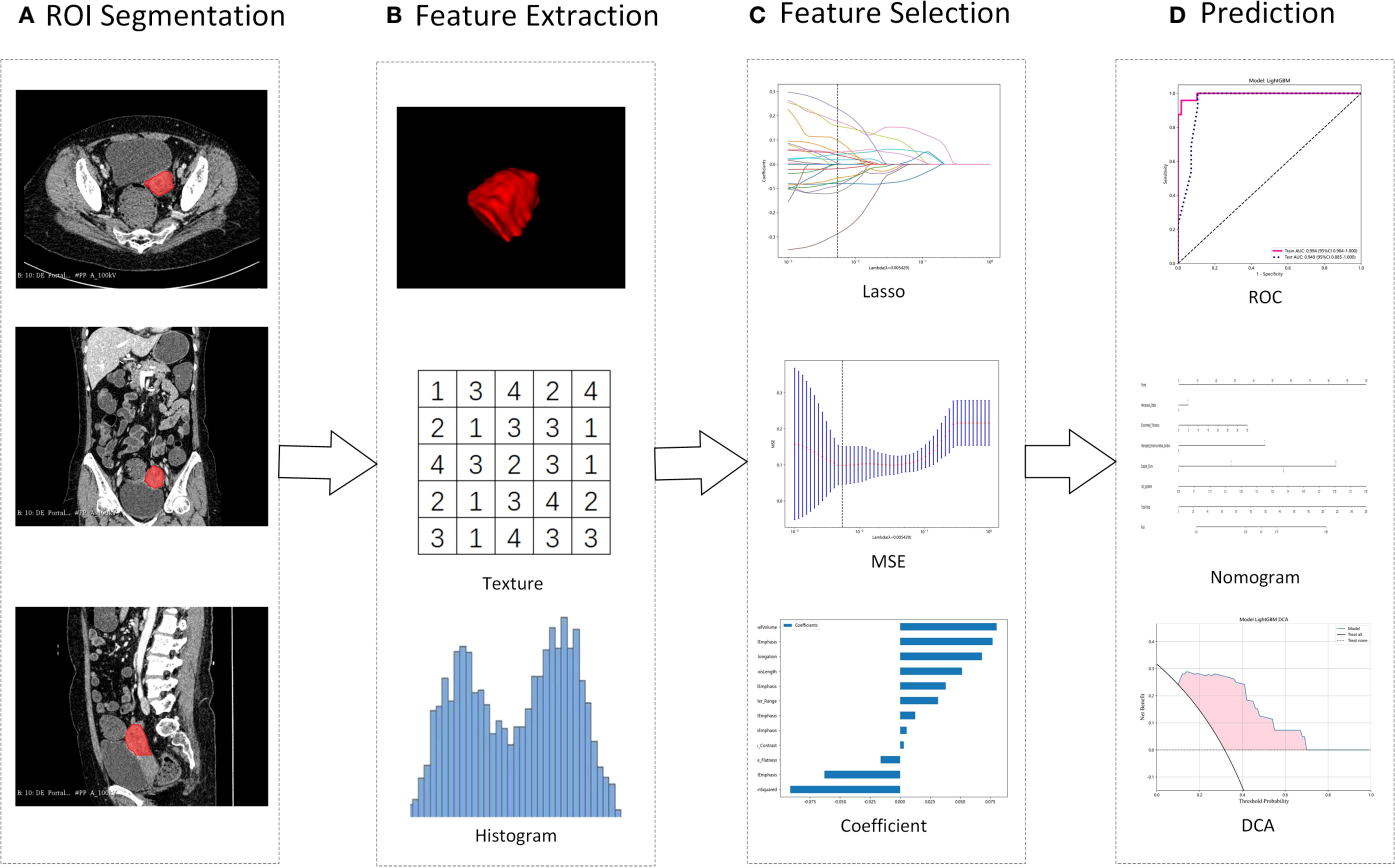
Illustration of the study pipeline. **(A)**, Intrauterine masses were segmented from contrast-enhanced CT as ROIs. **(B)**, From the ROI, 107 radiomics features were extracted, including geometry, intensity and texture. **(C)**, LASSO was used to select features, and Spearman’s rank correlation coefficient was used to calculate the correlation between features. **(D)**, Using the selected features, models were constructed to differentiate IVL and UM. ROI, regions of interest; LASSO, Least absolute shrinkage and selection operator; MSE, mean squared error; ROC, receiver operating characteristic curve. DCA, Decision Curve Analysis.

First, the robustness of the image features was evaluated. As the feature calculation depends on the ROI subregion contours, image features that are robust against ROI segmentation uncertainties were selected. Here, both test-retest analysis and interrater analysis were used to determine the feature robustness. Based on 35 patients randomly chosen from the discovery dataset, the test-retest analysis was performed, where for each patient, the tumor subregions were segmented twice by one rater. The dataset used for interrater analysis included another 35 randomly chosen patients, where for each patient, the ROI subregions were segmented by two raters independently. The features extracted from these multiple-segmented subregions were assessed using the intraclass correlation coefficient (ICC). Features with an ICC of ≥ 0.85 were considered robust against intra- and interrater uncertainties. Pipeline of radiomics in **Figure 2**.

#### 2.6.2 Spearman correlation

For features with high repeatability, Spearman’s rank correlation coefficient was also used to calculate the correlation between features (**Supplementary Figure 1** Spearman correlation of each feature), and one of the features with a correlation coefficient greater than 0.9 between any two features was retained. To retain the ability to depict features to the greatest extent, we use a stringent recursive deletion strategy for feature filtering; that is, the feature with the greatest redundancy in the current set is deleted each time.

#### 2.6.3 LASSO and radiomics signature

**Figure 3:**
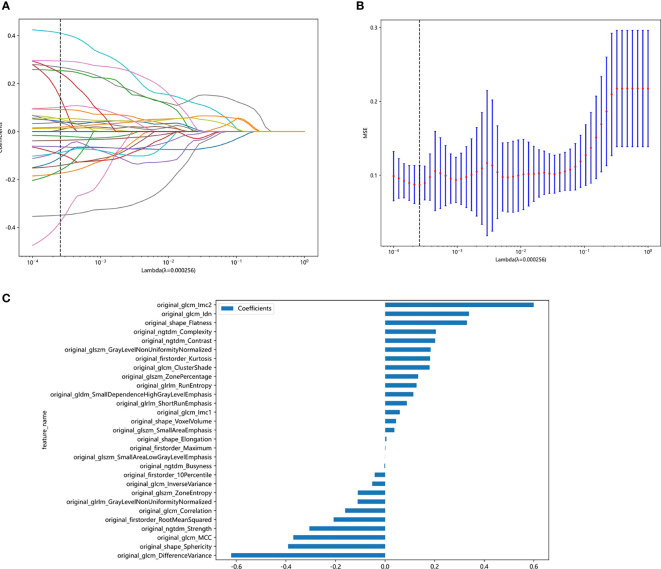
Figures of logistic LASSO regression. **(A)**, Lasso path plot of the model in the training dataset. **(B)**, Cross-validation plot for the penalty term. **(C)**, Spearman correlation coefficients between features were calculated, and 27 features with correlations were retained.

The least absolute shrinkage and selection operator (LASSO) Cox regression model was used on the discovery dataset for signature construction. Depending on the regulation weight λ, LASSO shrinks all regression coefficients toward zero and sets the coefficients of many irrelevant features exactly to zero. To find an optimal λ, 10-fold cross validation with minimum criteria was employed, where the final value of λ yielded the minimum cross validation error (**Figure 3**). The retained features with nonzero coefficients were used for regression model fitting and combined into a radiomics signature. Subsequently, we obtained a radiomics score (Rad-score) for each patient by a linear combination of retained features weighed by their model coefficients. The Python scikit-learn package was used for LASSO regression modeling. The histogram of the Rad-score is shown in **Figure 3**.

### 2.7 Clinical factor model construction

Age, body mass index (BMI), weight, height, symptoms, reproductive history, menopausal history, estrogen receptor (ER) status, progesterone receptor (PR) status, and diabetes were selected as clinical factors for the IVL and LM groups and analyzed for differences between groups. The selected clinical factors were fed into the LightGBM model for clinical signature building.

### 2.8 Radiomics model construction

After Lasso feature screening, we input the final features into the LightGBM model for risk model construction. Here, we adopt 3-fold cross verification to obtain the final radiomics signature. Receiver operating characteristic (ROC) curves were plotted to assess the diagnostic performance of the predictive models, and the corresponding area under the curve (AUC), diagnostic accuracy, sensitivity, specificity, positive predictive value (PPV), and negative predictive value (NPV) were analyzed.

### 2.9 Construction of the nomogram

Furthermore, to assess the incremental prognostic value of the radiomics signature to the clinical risk factors intuitively and efficiently, a radiomics nomogram was presented on the validation dataset. The nomogram combined the radiomics signature and the clinical risk factors based on logistic regression analysis. To compare the agreement between the IVL prediction of the nomogram and the actual observation, the calibration curve (Hosmer−Lemeshow H test) was calculated. The AUC was calculated simultaneously for the training and test groups to quantify the discriminability of the nomogram. The discriminability of the model was tested using the Delong test. Finally, decision curve analysis (DCA) was used to assess the clinical utility of this nomogram by quantifying the net benefit of the training and test sets of the combined model at different threshold probabilities.

### 2.10 Statistical analysis

The Python statamodels (version 0.13.2) package was used to perform statistical analysis, and a p value < 0.05 was considered statistically significant. We analyzed the differences between the IVL and uterine LM groups using Student’s t test or Mann−Whitney U tests for continuous variables; the chi-square test or Fisher’s exact test was applied for categorical variables.

## 3 Results

### 3.1 Patient characteristics

A total of 124 patients, including 37 IVL and 87 LM patients, were included in our study. Patients were divided into a training set (82 patients) and an independent test set (42 patients) based on treatment duration. A pathologist reviewed the pathological data. All patients underwent surgical treatment; there were 18 (48.6%) patients with IVL and 44 (50.6%) patients with LM in the training group and 19 (51.4%) patients with IVL and 43 (49.4%) patients with LM in the test group. The characteristics of the patients in the cohort are shown in **Table 1**. **Table 1** summarizes the patients’ baseline characteristics and postoperative pathological findings in the training and validation sets. The comparison of BMI, weight, height, ER, PR, diabetes, and fertility history showed no significant difference between the two groups and within each group (p>0.05), ensuring a reasonable classification. Significant differences between the cohorts were found in symptoms, menopause history, age, mass size, hypertension, and history of surgery (p<0.05).

**Table 1:** Demographic and clinical characteristics of study populations.

Characteristic	Total (n=124)	IVL (n=37)	UM (n=87)	p-value
**Age**	46.51 ± 8.17	43.32 ± 8.09	47.87 ± 7.86	0.004
**BMI**	24.01 ± 3.95	25.01 ± 3.03	23.59 ± 4.22	0.069
**Weight (kg)**	61.45 ± 10.93	63.82 ± 8.87	60.43 ± 11.59	0.114
**height (cm)**	159.89 ± 5.27	159.68 ± 4.94	159.98 ± 5.43	0.768
**Symptoms**	68 (0.5484)	35 (0.9459)	33 (0.3793)	<0.001
**Reproductive history**	114 (0.9194)	34 (0.9189)	80 (0.9195)	0.991
**menopause**	40 (0.3226)	5 (0.1351)	35 (0.4023)	0.003
**ER-** Positive	100 (0.8065)	33 (0.8919)	67 (0.7701)	0.118
**PR-** Positive	123 (0.9919)	36 (0.9730)	87 (1.0000)	0.126
**Hypertension**	28 (0.2258)	4 (0.1081)	24 (0.2759)	0.041
**Diabetes**	17 (0.1371)	5 (0.1351)	12 (0.1379)	0.967

BMI, body mass index; ER, estrogen receptor; PR, progesterone receptor.

### 3.2 Feature selection and radiomics signature development

Features with an ICC of ≥ 0.85 were considered robust against intra- and interrater uncertainties. After robustness evaluation, 62 categories out of the initial 108 image features remained. Spearman correlation coefficients between features were calculated, and features with correlations were retained (**Supplementary Table 1**). As shown in **Figure 3**, 29 features of nonzero coefficients were selected to establish the Rad-score with a LASSO logistic regression model (λ = 0.005429). The formula used to calculate the Rad-score is described in the **Supplementary Materials** (**Supplementary Table 1**).

### 3.3 Clinical factor model

**Figure 4:**
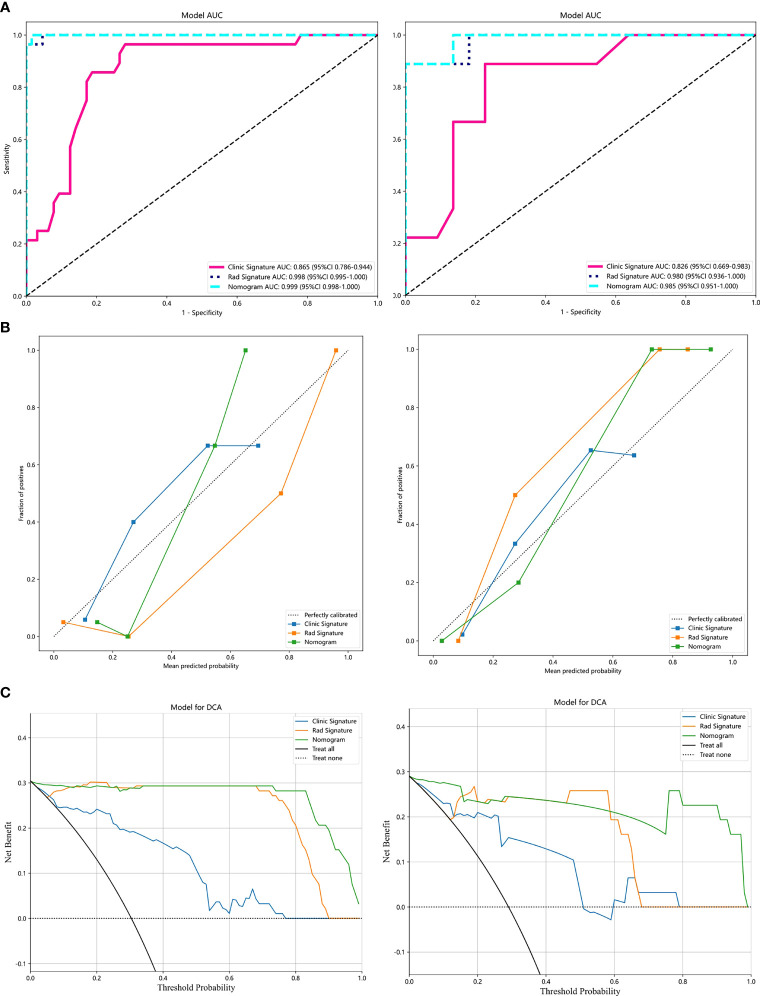
Results of the LightGBM models: **(A)**, Receiver operator characteristic curves of the 3 LightGBM models for identifying patients with IVL and uterine LM in the training and test datasets. **(B)**, The calibration curve of the 3 models. **(C)**, The decision curve analysis (DCA) of the three models of the training and test datasets.

Analysis of differences between groups showed that symptoms, menopausal history, and age were independent clinical risk factors for IVL (**Table 1**). A clinical signature was composed of three factors selected, namely, symptoms, menopausal history, and age. In the training group, the AUC value of the radiomics model was 0.865 (95% CI 0.786–0.944); in the test group, the AUC value of the model was 0.826 (95% CI of 0.669–0.983) (**Table 2**, **Figure 4A**).

**Table 2:** Main consequence of 3 models based on Light Gradient Boosting Machine(LightGBM)algorithm.

Model	Dataset	Accuracy	AUC	95% CI	Sensitivity	Specificity	PPV	NPV	Precision	Recall	F1	Threshold
Clinical	Train	0.793478	0.865	0.786 – 0.944	0.964286	0.718750	0.600000	0.978723	0.600000	0.964286	0.739726	0.240545
	Test	0.806452	0.826	0.669 – 0.983	0.888889	0.772727	0.615385	0.944444	0.615385	0.888889	0.727273	0.268398
Radiomics	Train	0.989247	0.998	0.995 - 1.000	0.964286	1.000000	1.000000	0.984848	1.000000	0.964286	0.981818	0.730348
	Test	0.967742	0.980	0.936 - 1.000	0.888889	0.772727	0.615385	0.944444	0.615385	0.888889	0.727273	0.268398
Combined	Train	0.999442	0.999	0.998 – 1.000	1.000000	0.984375	0.965517	1.000000	0.965517	1.000000	0.982456	0.232776
	Test	0.967742	0.985	0.951 - 1.000	0.888889	1.000000	1.000000	0.956522	1.000000	0.888889	0.941176	0.586624

AUC, Area Under Curve; 95% CI, 95% Confidence Interval; NPV, Negative predictive value; PR, Progesterone receptor; PPV, Positive predictive value; F1, F1 Score.

### 3.4 Diagnostic performance of radiomics features

Our results show that the radiomics features have good predictive performance for both the training and test sets. The AUCs of the radiomics model were significantly larger than those of the clinical model in both the training dataset (AUC=0.998 95% CI: 0.995-1.000) and the validation dataset (AUC=0.98; 95% CI: 0.936-1.000) (**Table 2**, **Figure 4A**).

### 3.5 Combined models and radiomics nomogram

A combined model was developed by integrating the Rad-score and clinical predictors. A good performance was shown for the combined nomogram model in both the training dataset (AUC = 0.999 95% CI: 0.998-1.000) and the validation dataset (AUC = 0.985; 95% CI: 0.951-1.000) (**Table 2**, **Figure 4A**). The diagnostic accuracy, sensitivity, specificity, PPV, NPV, precision and recall of the three models are also demonstrated in **Table 2**.

The calibration curve showed that the IVL predicted by the combined model was very close to the actual results in both datasets (**Figure 4B**). The DCA also revealed the improvement in the combined model in both datasets (**Figure 4C**). This showed that when the threshold probability was between 1% and 99%, the combined model was more beneficial than the Rad-score and clinical models.

**Figure 5:**
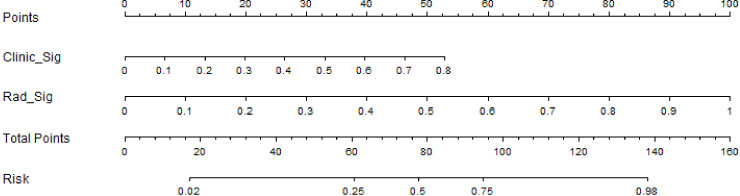
Nomograph based on the combined model.

We also developed a nomogram to visualize the model for the combination (**Figure 5**). In the nomogram, points for each variable can be added to the corresponding axis to determine the risk of IVL. A higher total score is associated with a greater risk of IVL.

According to the DeLong test, the AUCs of the nomogram-based models in the training and test sets were significantly different from those of the clinical model (P=0.046) (**Supplementary Table 2**). Therefore, we found that the nomogram method performed well on both sets of data. Furthermore, the Hosmer−Lemeshow test showed no statistically significant difference between the training and testing subsets (p>0.05) (**Table 3**).

**Table 3:** Hosmer-Lemeshow test.

	Clinic Signature	Rad Signature	Nomogram
**UM**	0.099372	0.598583	0.046526
**IVL**	0.459798	0.913913	0.560273

Hosmer-Lemeshow test showed no statistically significant difference between the training and testing subsets (p>0.05)

## 4 Discussion

In this retrospective study, we constructed for the first time a comprehensive model incorporating the Rad-score, symptoms, menopausal history and age and established a preoperative distinction between IVL and LM based on contrast-enhanced CT images.

The combined model consisting of radiomic features and clinical factors exhibited the best discriminatory ability and fit, indicating a good diagnostic performance. The AUC values of the model were 0.999 and 0.985 in the training and test groups, respectively.

LM is the most common uterine neoplasm in gynecology, with a prevalence of up to 20-30% in women of childbearing age. It has typical imaging features and clinical manifestations, and the radiological diagnosis of classic LM is definitive (9, 10). Occasionally, however, LM with rare growth patterns occurs, mostly in women of reproductive age, and IVL is one type of LM with an unusual growth pattern that presents as serpentine growth within the inferior vena cava (IVC) and genital veins and may spread to the right atrium (RA), making its identification clinically and radiologically more challenging (4). Worldwide, fewer than 300 cases of IVL and fewer than 100 cases of cardiac involvement have been reported. The imaging features of IVL are unclear and are often misdiagnosed preoperatively. It is mostly evaluated clinically using multimodal imaging techniques such as echocardiography, contrast-enhanced CT and MRI, which can provide important information revealing the extent and location of the mass and are used to determine surgical options (11). Echocardiography can assess the extension of the tumor into the RA, and CT and MRI can show the continuity of intraluminal tumor growth from the pelvic veins. It has been suggested that MRI is a particularly valuable imaging technique for the preoperative evaluation of IVL, which in the inferior vena cava looks similar to a sieve on axial images and to a sponge on T2-weighted images with several fissures parallel to the IVL, which may lead to turbulent blood flow (12–15). However, MRI has poor spatial resolution and is time-consuming and unsuitable for patients with metal in their bodies. Enhanced CT can produce multilevel enhanced CT data in a short period, directly displaying the full extent of the tumor, with a sponge and sieve appearance similar to MRI (16). In addition, the combined scan of the chest, abdomen and pelvis can clearly show the changes in the uterus and the extent of tumor invasion (17, 18). According to previous reports in the literature, radiologists are prone to misdiagnose IVL located in the venous system or RA as an occupying lesion, mainly leiomyosarcoma, RA myxoma, endometrial stromal sarcoma, and intravenous thrombosis (19, 20). However, these patients usually do not have a history of LM. It is almost impossible for radiologists to distinguish IVL confined to parauterine veins without distant venous system invasion, and in the early stages, LM is difficult to distinguish completely from IVL clinically and radiologically.

Previous studies did not find significant differences between IVL and LM in terms of histomorphology and immunophenotype, such as both expressing ER and PR and smooth muscle cell markers, and no elevated proliferation index or nuclear division number was found, suggesting that both have more of the same intrinsic molecular basis. Our data and recent reports suggest that IVL accounts for approximately 1% of LM surgical specimens and its incidence is increasing. Some providers have an inadequate understanding of IVL, therefore, there are more missed diagnoses and its incidence is seriously underestimated (21–23). Some scholars compared the transcriptomic data of IVL and LM and found that antiapoptosis and angiogenesis-related genes may be novel biomarkers of IVL, indicating that IVL is very different from LM on a molecular and genetic basis. Further analysis of their gene expression profiles revealed that IVL and LM share some molecular genetic features and that IVL has a similar expression profile to leiomyosarcoma, further supporting that IVL has a quasi-malignant behavior and is not a distinct variant of LM (24, 25). However, these molecular genetic features are not independent predictors, and although they are associated with the occurrence of IVL, they do not distinguish IVL from LM.

By extracting high-dimensional imaging features from different modality images and mining the data, radiomics can be used for molecular typing of tumors, differential diagnosis, treatment option selection, efficacy detection and prognosis assessment (6).

These high-dimensional features are indistinguishable by the human eye and contain biological information determined by genes, proteins and tissue microcomponents, which radiomics can measure (26, 27). A radiomic model with features extracted from a ROI containing the whole uterus was shown to have good diagnostic performance for uterine fibroids and uterine sarcomas with an AUC of 0.83 (7). Some studies have used radiomic features to distinguish uterine sarcomas from atypical fibroids, showing better diagnostic efficacy than MRI features alone. Radiologists achieved an AUC of 0.752 for MRI-based diagnostic efficacy, and the radiomic model achieved an AUC of 0.830 (28). One study established an MRI-based radiomic nomogram for detecting deep myometrial invasion in early-stage endometrioid adenocarcinoma, showing superior diagnostic accuracy to radiologists, with an AUC of 0.883 (29). This suggests that radiomic methods can better predict and differentiate the type of uterine tumors compared to traditional clinical features. However, there are no relevant radiomic studies to better differentiate and distinguish uterine smooth muscle tumors with unusual growth patterns, which are often rare and require multiple imaging techniques to aid in the differential diagnosis.

In our study, the nomogram was constructed using the Radscore and contrast-enhanced CT with radiological methods. The Radscore is described as the probability of principal component analysis calculated from the radiomic signature, which is constructed based on sixteen selective radiomic features. The AUCs for predicting the radiomic features of IVL were 0.998 (training group) and 0.980 (test group). Nomograms constructed from radiological and clinical features show good discrimination between IVL and LM. The AUC values of the training and test groups were 0.999 (95% CI: 0.998–1.000) and 0.985 (95% CI: 0.951-1.000), respectively. The results showed that the nomogram effectively predicted IVL in both the training and validation groups, exceeding the predictive accuracy of the radiomics and clinical models. The decision curve suggests that patients could benefit more from using the radiological nomogram in this study if they have a threshold probability of 1% to 99%. The combined model has better predictive performance than clinical factors or radiological features alone. The model is clear, simple, and easy to understand, which makes it more suitable for clinical application.

In the analysis of clinical factors between the IVL and LM groups, there were significant differences in age, symptoms, and menopausal history, so we introduced these factors into the clinical model and they demonstrated some predictive capacity. IVL often has no specific symptoms before causing cardiac insufficiency, and its clinical manifestations are usually related to the scope and size of the tumor (25). In clinical practice, we have found that IVL extending to the extrauterine venous system often accompanies large pelvic LM and causes related symptoms. However, only a tiny percentage of LMs develop at unusual locations beyond the uterus. All IVL cases occur in women, and the literature reports that the mean age of onset is 47 years; 90% are premenopausal, and 64% have uterine fibroids or a history of hysterectomy (30). The mean age of the cases in this group was 43.3 years; patients with a history of menopause in IVL were significantly younger than those in the LM group, which is similar to the literature.

This study still has some limitations. First, the sample size was relatively small, and it was a single-center study because the study population was a rare disease. Second, this study was retrospective, which may lead to patient selection bias. Third, manual ROI segmentation has inherent inter- and intra-observer differences. Fourth, we only built a radiomic model based on enhanced CT without using other imaging, so it is impossible to gage the quality of each image. In the future, we will include more patients and make further technical improvements, such as fully automated image segmentation, deep learning and multiparametric modeling, to explore more accurate radiological diagnoses.

## 5 Conclusion

In conclusion, our study confirmed that a radiomics nomogram model and radiomics signature based on contrast-enhanced CT can help differentiate between IVL and LM patients and predict whether IVL will invade the extrauterine vessels when it is still confined to the uterus to guide clinical treatment.

## Data availability statement

The datasets presented in this article are not readily available because this dataset is not publicly available due to patient privacy. Requests to access the datasets should be directed to Jiang Shao, esan2002@vip.sina.com.

## Ethics statement

Written informed consent was obtained from the individual(s) for the publication of any potentially identifiable images or data included in this article.

## Author contributions

All authors contributed significantly to this work; all authors revised the manuscript and gave final approval of the manuscript to be submitted. JS and BL: Conceptualization, Methodology. CW, KS and NC: Data curation, Writing-Original draft preparation. JC, FD, XY, and ZZ: Visualization, Investigation. ZL: Supervision. JW, YF, XL, JY, YY: Software, Validation. YZ, KS and BL: Writing- Reviewing and Editing.
