# Supplementary material for: A contrast-enhanced CT-based radiomic nomogram for the differential diagnosis of intravenous leiomyomatosis and uterine leiomyoma
Source: Front Oncol. 2023 Aug 23;13:1239124. doi: 10.3389/fonc.2023.1239124 (PMC10482096; doi:10.3389/fonc.2023.1239124)
Supplement: Supplementary file 1 [file DataSheet_1.zip › Table 1.DOCX]

**Supplementary table 1：**The formula used to calculate the Rad score

| rad_score = 0.29908628206216237 -0.042229 * original_firstorder_10Percentile +0.181523 * original_firstorder_Kurtosis +0.001924 * original_firstorder_Maximum -0.207499 * original_firstorder_RootMeanSquared +0.179380 * original_glcm_ClusterShade -0.161480 * original_glcm_Correlation -0.621321 * original_glcm_DifferenceVariance +0.338646 * original_glcm_Idn +0.059245 * original_glcm_Imc1 +0.599407 * original_glcm_Imc2 -0.052303 * original_glcm_InverseVariance -0.370302 * original_glcm_MCC +0.113921 * original_gldm_SmallDependenceHighGrayLevelEmphasis -0.111292 * original_glrlm_GrayLevelNonUniformityNormalized +0.126911 * original_glrlm_RunEntropy +0.087709 * original_glrlm_ShortRunEmphasis +0.183881 * original_glszm_GrayLevelNonUniformityNormalized +0.037562 * original_glszm_SmallAreaEmphasis -0.000516 * original_glszm_SmallAreaLowGrayLevelEmphasis -0.110207 * original_glszm_ZoneEntropy +0.133445 * original_glszm_ZonePercentage -0.002947 * original_ngtdm_Busyness +0.204609 * original_ngtdm_Complexity +0.201887 * original_ngtdm_Contrast -0.305561 * original_ngtdm_Strength +0.005702 * original_shape_Elongation +0.330350 * original_shape_Flatness -0.391530 * original_shape_Sphericity +0.044138 * original_shape_VoxelVolume |
| --- |
