# Supplementary material for: A contrast-enhanced CT-based radiomic nomogram for the differential diagnosis of intravenous leiomyomatosis and uterine leiomyoma
Source: Front Oncol. 2023 Aug 23;13:1239124. doi: 10.3389/fonc.2023.1239124 (PMC10482096; doi:10.3389/fonc.2023.1239124)
Supplement: Supplementary file 1 [file DataSheet_1.zip › Table 2.DOCX]

**Supplementary table 2：**Delong test

| cohort | Nomogram Vs Clinic | Nomogram Vs Rad |
| --- | --- | --- |
| train | 0.000783 | 0.412977 |
| test | 0.045865 | 0.479500 |
