# Supplementary figures and images for: A contrast-enhanced CT-based radiomic nomogram for the differential diagnosis of intravenous leiomyomatosis and uterine leiomyoma
Source: Front Oncol. 2023 Aug 23;13:1239124. doi: 10.3389/fonc.2023.1239124 (PMC10482096; doi:10.3389/fonc.2023.1239124)

**Supplementary figure 1：heatmap map and** spearman correlation of each feature


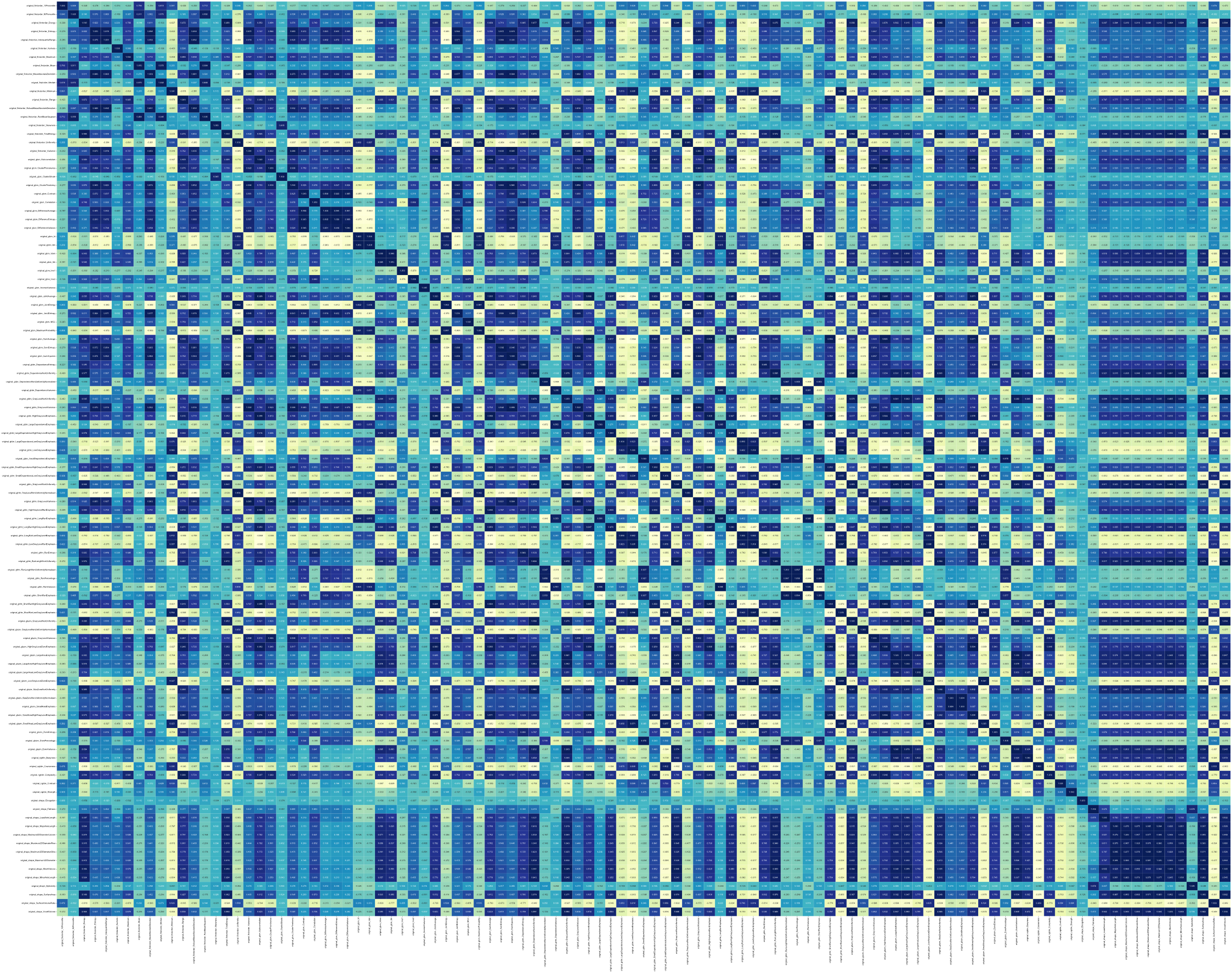

Supplement: Supplementary file 1 [file DataSheet_1.zip › Figure 1.DOCX]
